# PS77: a novel peptide with α-helical structure for targeted anti-inflammatory therapy in biomaterials design

**DOI:** 10.1007/s12026-025-09663-0

**Published:** 2025-07-24

**Authors:** Zhengyi Lin, Haiyi Zhao, Haojie Lin, Lanni Song, Xuechen Tian, Siew Woh Choo

**Affiliations:** 1 College of Science, Mathematics and Technology, Wenzhou-Kean University, 88 Daxue Road, Ouhai, Wenzhou, 325060 Zhejiang Province China; 2 Wenzhou Municipal Key Laboratory for Applied Biomedical and Biopharmaceutical Informatics, Wenzhou-Kean University, 88 Daxue Road, Ouhai, Wenzhou, 325060 Zhejiang Province China; 3 Zhejiang Province-Malaysia International Joint Laboratory for Modern Agriculture and Microbial Innovation, Wenzhou-Kean University, 88 Daxue Road, Ouhai, Wenzhou, 325060 Zhejiang Province China; 4 Dorothy and George Hennings College of Science, Mathematics and Technology, Kean University, 1000 Morris Ave, Union, 07083 NJ USA

**Keywords:** Anti-inflammatory peptide, Synthetic peptide PS77, TNF-α-induced inflammation, BMP and TGF-β signaling pathways, Transcriptomics

## Abstract

Chronic inflammation underlies many diseases, posing challenges in therapeutic management due to the limitations and side effects of current treatments and necessitating novel therapeutic solutions. Here, we introduce PS77, a novel α-helical peptide derived from Squama Manitis, a Traditional Chinese Medicine, and unveil its remarkable anti-inflammatory properties, potentially revolutionizing biomaterials design for targeted anti-inflammatory therapies. An in vitro TNF-α-induced inflammatory model in human keratinocytes (HaCaT cells) was used to demonstrate PS77’s significant impact. We demonstrated that PS77 significantly reduced IL-8 and MMP-3 expression, indicating potent anti-inflammatory activity without cytotoxicity to normal cells. Transcriptomic analysis further elucidated PS77’s mechanism of action, revealing significant modulation of 265 genes (137 upregulated and 128 downregulated), with a particular focus on the downregulation of genes within the BMP and TGF-β signaling pathways—key players in inflammation. Moreover, PS77 regulated several inflammation-associated genes, including CHRNA7, CXCR5, RXRG, KRT76, IL12RB2, and COLEC11, underscoring its comprehensive anti-inflammatory effects. This study not only highlights PS77’s therapeutic potential as a biomaterial for treating inflammatory diseases but also paves the way for further research into its mechanisms and applications in biomedicine. By leveraging the novel biomaterial properties of PS77, this research may contribute to the development of targeted and efficient anti-inflammatory therapies, marking a significant advance in the field of biomaterials and offering a promising avenue for inflammation management.

**Supplementary Information:**

The online version contains supplementary material available at 10.1007/s12026-025-09663-0.

## Introduction

Inflammation is a vital protective mechanism against foreign pathogens and injury [1]. However, dysregulated or chronic inflammation can lead to various diseases, such as rheumatoid arthritis [2, 3], inflammatory bowel disease [4], and cancer [5, 6]. Current anti-inflammatory therapies, including non-steroidal anti-inflammatory drugs (NSAIDs) and corticosteroids, often have limited efficacy and are associated with adverse side effects [7, 8]. Therefore, there is an urgent need to develop novel anti-inflammatory agents with improved efficacy and safety profiles.

In recent years, the field of biomaterials has increasingly intersected with anti-inflammatory therapies, heralding a new era of therapeutic interventions [9, 10]. Current biomaterial-based strategies for inflammation management include drug delivery systems, scaffolds for tissue engineering, and implants designed to modulate immune responses. Despite these advancements, challenges such as non-specificity, limited biocompatibility, and inadequate targeting capabilities persist, underscoring the pressing need for innovative biomaterials that offer targeted, efficient, and biocompatible solutions for inflammation management [11, 12]. In this context, peptide-based biomaterials, characterized by their specificity, minimal toxicity, and versatility, have emerged as promising candidates [13]. Many peptides with anti-inflammatory properties have been identified from natural sources, such as plants, animals, and microorganisms [14]. Traditional Chinese Medicine (TCM) has been a valuable source of bioactive compounds, including peptides, with potential therapeutic applications [15].

Squama Manitis, a TCM derived from pangolin scales, has been used to treat various diseases, including inflammation and cancer [3, 16]. Lv et al. demonstrated that Squama manitis extract exhibits significant anti-inflammatory activity in arthritis (RA) rat model. However, its anti-inflammatory compounds remain unknown. We recently discovered a novel peptide, PS77 (China Invention Patent Number: 202110622991.7), from Squama Manitis and investigated its anti-inflammatory effects using an in vitro TNF-α-induced inflammatory model in human keratinocytes (HaCaT cells).

TNF-α is a key pro-inflammatory cytokine that plays a central role in the pathogenesis of various inflammatory diseases [17]. HaCaT cells are widely used as an in vitro model to study skin inflammation and to evaluate the efficacy of anti-inflammatory agents [18]. By employing this model, we aimed to elucidate the anti-inflammatory mechanisms of PS77 and explore its potential as a novel therapeutic agent for inflammatory diseases.

In this study, we synthesized PS77 using solid-phase peptide synthesis and evaluated its effects on cell viability and the expression of pro-inflammatory cytokines IL-8 and MMP-3 in TNF-α-stimulated HaCaT cells. Furthermore, we performed transcriptomic analysis to identify the differentially expressed genes and signaling pathways modulated by PS77 treatment. Our findings provide new insights into the anti-inflammatory mechanisms of PS77 and highlight its potential as a novel peptide-based biomaterial for the treatment of inflammatory diseases, aligning with the principles of precision medicine and biocompatibility in the biomaterials field.

## Methods and materials

### Peptide synthesis

The peptide PS77 was synthesized using solid-phase peptide synthesis (SPPS). Briefly, a modified resin was used as the solid support, and the reactor was treated with a 20% piperidine/dimethylformamide (DMF) solution for 20 min to swell the resin. The solution was filtered off, and the resin was washed with DMF three times. Fmoc deprotection was carried out, and the coupling of amino acids was performed using a prepared amino acid solution and diisopropylcarbodiimide (DIC)/DMF solution. The success of Fmoc deprotection and amino acid coupling was monitored using a colorimetric test with detection reagents A and B. The peptide structure was predicted and visualized using the Ressource Parisienne en Bioinformatique Structurale (RPBS) web server[19].

### Cell culture and human inflammatory model

Human immortalized keratinocytes (HaCaT) cells were seeded and cultured in Dulbecco's modified Eagle's medium (DMEM; Gibco, USA) supplemented with 10% (v/v) fetal bovine serum (Gibco, USA) and 1% (v/v) penicillin/streptomycin at 37 °C in a humidified atmosphere with 5% CO_2_. The in vitro inflammatory model was established by treating HaCaT cells with human tumor necrosis factor-α (TNF-α). Cell viability was measured using the Cell Counting Kit-8 (CCK-8) assay, and TNF-α expression was quantified using an enzyme-linked immunosorbent assay (ELISA) with a microplate reader. The statistical analysis was performed by GraphPad 6.0 software (GraphPad, San Diego, USA) by applying a t-test. All the results were expressed based on the mean ± standard error of mean (SEM).

### Cell viability and toxicity assay

HaCaT cells were seeded in 96-well plates at a density of 3 × 10^4^ cells/mL and treated with PS77 at concentrations of 0.1, 1, 10, and 100 μg/mL for 24 h. Cell viability was determined using the CCK-8 assay kit according to the manufacturer’s instructions. Absorbance was measured at 450 nm using a microplate reader (Thermo Fisher, USA), and cell viability was calculated as a percentage relative to the untreated control. The optimal peptide concentration for subsequent experiments was selected based on the cell viability assay. All the statistical analysis was done using Graphpad Prism (USA).

### ELISA assay

HaCaT cells were seeded in 96-well plates at a density of 3 × 10^4^ cells/mL and treated with or without 0.1 μg/mL PS77 for 24 h, followed by the addition of 2 ng/mL TNF-α and further incubation for 12 h at 37 °C. The expression levels of matrix metalloproteinase-3 (MMP-3) and interleukin-8 (IL-8) were measured using ELISA kits (Cloud-Clone Corp, USA) according to the manufacturer’s protocol. One hundred microliters of each sample was added to the pre-coated 96-well based on the instruction manual. The detection reagent A was added to the samples and incubated for 1 h. Then after washing, 100 μL detection reagent B was added. According to the manual, 90 μL of substrate solution and 50 μL stop solution was added to the sample and the absorbance was obtained under 450 nm.

### RNA extraction

Total RNA was extracted from HaCaT cells treated with or without 0.1 μg/mL PS77 using TRIzol® reagent (Invitrogen, USA) according to the manufacturer's instructions. Briefly, both groups were pre-treated with TNF-α. The volume of the reagent was adjusted to the TRIzol® treated sample according to the manufacturer’s instructions. Tubes containing TRIzol®-chloroform fractions were centrifuged at 12,000 × *g* for 5 min at 4 °C and the supernatant was removed. Subsequently, chloroform was added to emulsify. The tubes were mixed upside down and centrifuged at 2,000 × *g* for 15 min at 4 °C. The supernatant was transferred to a 2-ml tube for RNA extraction. The RNA was precipitated with isopropanol, then mixed, and incubated for 10 min at room temperature. The samples were centrifuged at 12,000 × *g* for 10 min and the supernatant was discarded. Later, the RNA pellet was washed in 75% ethanol, incubated at room temperature for 5 min and stored at −80 °C for the later sequencing. RNA quality and quantity were assessed using a NanoDrop spectrophotometer (Thermo Fisher, USA) and an Agilent 2100 Bioanalyzer (Agilent Technologies, USA).

### Library preparation and whole-transcriptome sequencing

The library preparation and RNA sequencing were performed by GENEWIZ company (Suzhou, China). The illumina (San Diego, CA, USA) sequencing technology was used to conduct the RNA library preparation and sequencing. The isolation of Poly(A) mRNA was carried out based on Oligo(dT) beads. By using divalent cations at high temperatures, mRNA fragmentations were produced. To synthesize both first-strand cDNA and second-strand cDNA, random primers were employed. The resulting cDNA with double-stranded was meticulously repaired, including the addition of dA tails, and T-A ligation to introduce adapters at both ends. DNA Clean Beads were utilized to perform size selection of the adapter-ligated DNA. Subsequently, PCR amplification of each sample was conducted utilizing P5 and P7 primers, which were also employed for verification of the PCR products. For sequencing, a high-throughput Illumina Novaseq sequencing platform with a 2 × 150 paired-end (PE) configuration was utilized, strictly following the manufacturer's instructions.

### Read preprocessing and mapping

Raw sequencing reads were processed using Cutadapt (v1.9.1; phred cutoff: 20, error rate: 0.1, adapter overlap: 1 bp, min. length: 75, proportion of N: 0.1) to remove adapters and low-quality bases (< Q20) [20]. Clean reads were mapped to the human reference genome (GRCh38.p13) using HISAT2 (v2.0.1) [21]. Gene expression levels were quantified using HTSeq (v0.6.1) [22]. FPKM (Fragments Per Kilo bases per Million reads) method was used in this software to calculate the gene expression levels. Differentially expressed genes (DEGs) between the control and PS77-treated groups were identified using DESeq2 Bioconductor software (v.3.10), with a fold change ≥ 2 and *p*-value < 0.05 considered significant [23, 24]. Differential expression genes with positive fold change (FC ≥ 2) levels are defined as “up-regulated”; otherwise, the negative fold change (FC ≤  − 2) levels are considered “down-regulated.”

### Functional enrichment analysis

To examine the biological processes and pathways associated with DEGs, functional enrichment analysis was performed using the sets of significant DEGs using Gene Ontology (GO) and Kyoto Encyclopedia of Genes and Genomes (KEGG) pathway databases [25]. GOSeq (v1.34.1) [26] was used to identify GO terms that annotate a list of enriched genes with a significant Padj less than 0.05.

## Results

### Synthesis and characterization of peptide PS77

**Fig. 1:**
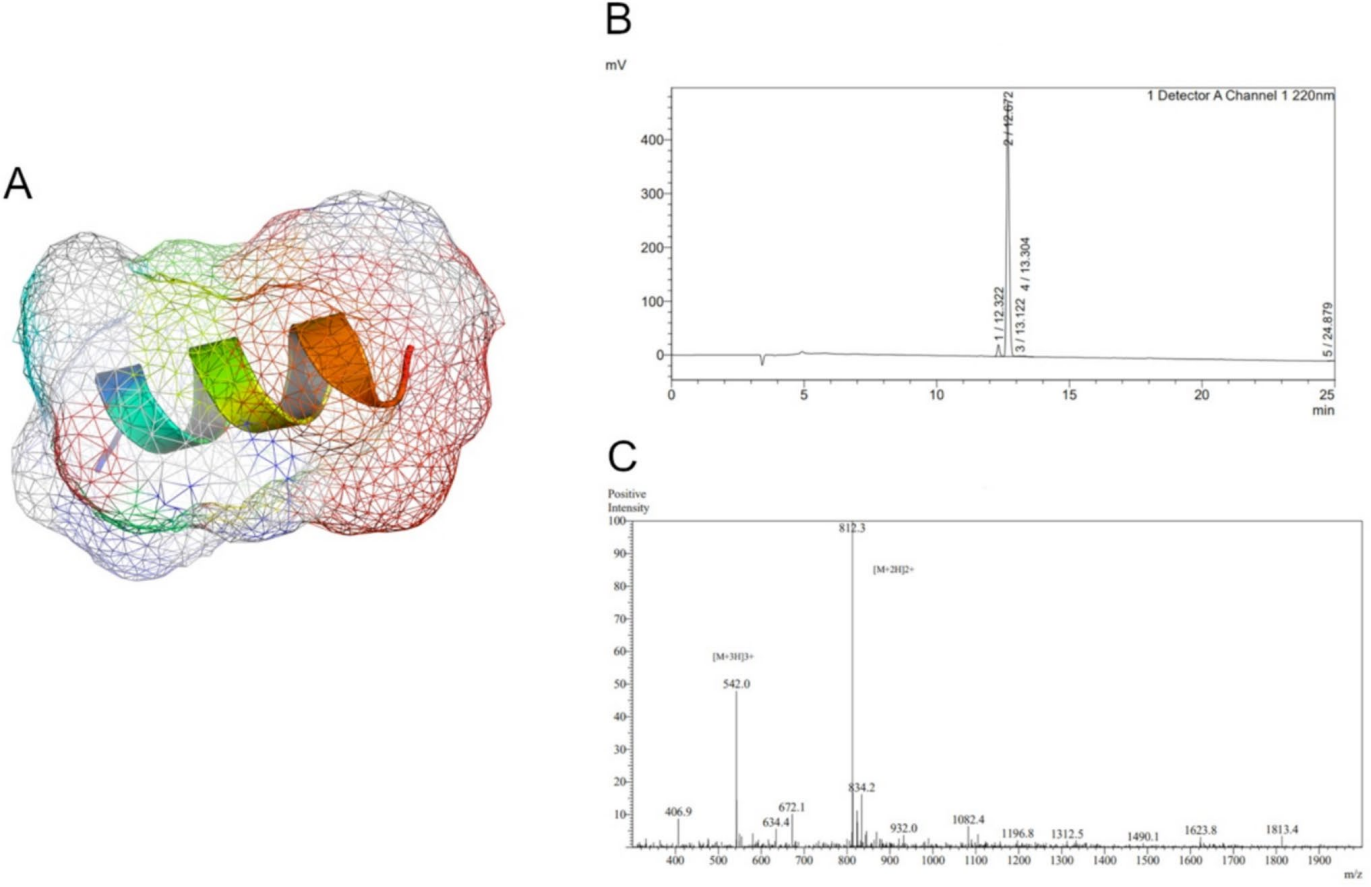
**A** The predicted 3D structure of PS77 with Van der walls forces. **B** HPLC spectrum and the corresponding peak intensities of PS77. It showed the chromatographic separation of the peptide sample, with the x-axis representing the retention time and the y-axis indicating the intensity or absorbance of the detected compounds. **C** MS spectrum and the corresponding peak intensities of PS77. The MS spectrum displayed the mass-to-charge ratio (m/z) of the peptide fragments on the x-axis and their relative abundance on the y-axis. The single and intense peak corresponds to the molecular weight of the PS77 peptide

PS77, a 13-amino acid peptide (molecular weight = 1622.92 Da; Supplementary Table 1), was chemically synthesized using solid-phase peptide synthesis (SPPS). The peptide's α-helical structure, which is a common secondary structure in peptides and proteins, was confirmed (Fig. 1A). The α-helix is stabilized by hydrogen bonds between the backbone amino and carboxyl groups, and it is often associated with biological activity and stability. The presence of an α-helical structure suggests that PS77 may have a specific function and could potentially interact with target molecules or receptors. High-performance liquid chromatography (HPLC) revealed a single, sharp peak with a high intensity and with the purity of 95.3%, indicating that the PS77 peptide is highly pure and homogeneous (Fig. 1B). This high purity is essential for accurate characterization of the peptide’s properties and biological effects. Mass spectrometry (MS) analysis verified molecular weight (1622.92 Da) of PS77 (Fig. 1C). The absence of other significant peaks indicates that the peptide has a well-defined molecular mass and is not degraded or modified. The MS data confirms the identity and integrity of the PS77 peptide. Taken all together, the combination of 3D structure prediction, HPLC, and MS analyses provides a comprehensive characterization of the PS77 peptide. The predicted α-helical conformation suggests a potential biological function, while the HPLC and MS data confirm the high purity and integrity of the peptide sample. These results support the use of PS77 in further biological studies and its potential development as a therapeutic agent.

Furthermore, the α-helical structure of PS77 is pivotal in biomaterial design, conferring enhanced specificity in targeting inflammatory pathways, which is crucial for developing targeted therapies with minimal off-target effects. This structural configuration also contributes to the peptide’s stability and biocompatibility, essential attributes for any biomaterial intended for therapeutic use, ensuring that it remains active and well-tolerated within the biological environment. Moreover, these properties make PS77 an ideal candidate for integration into sophisticated delivery systems or scaffolds for tissue engineering, facilitating localized therapy at inflammation sites while minimizing systemic exposure and potential side effects.

### Establishing TNF-α-induced inflammatory model

**Fig. 2:**
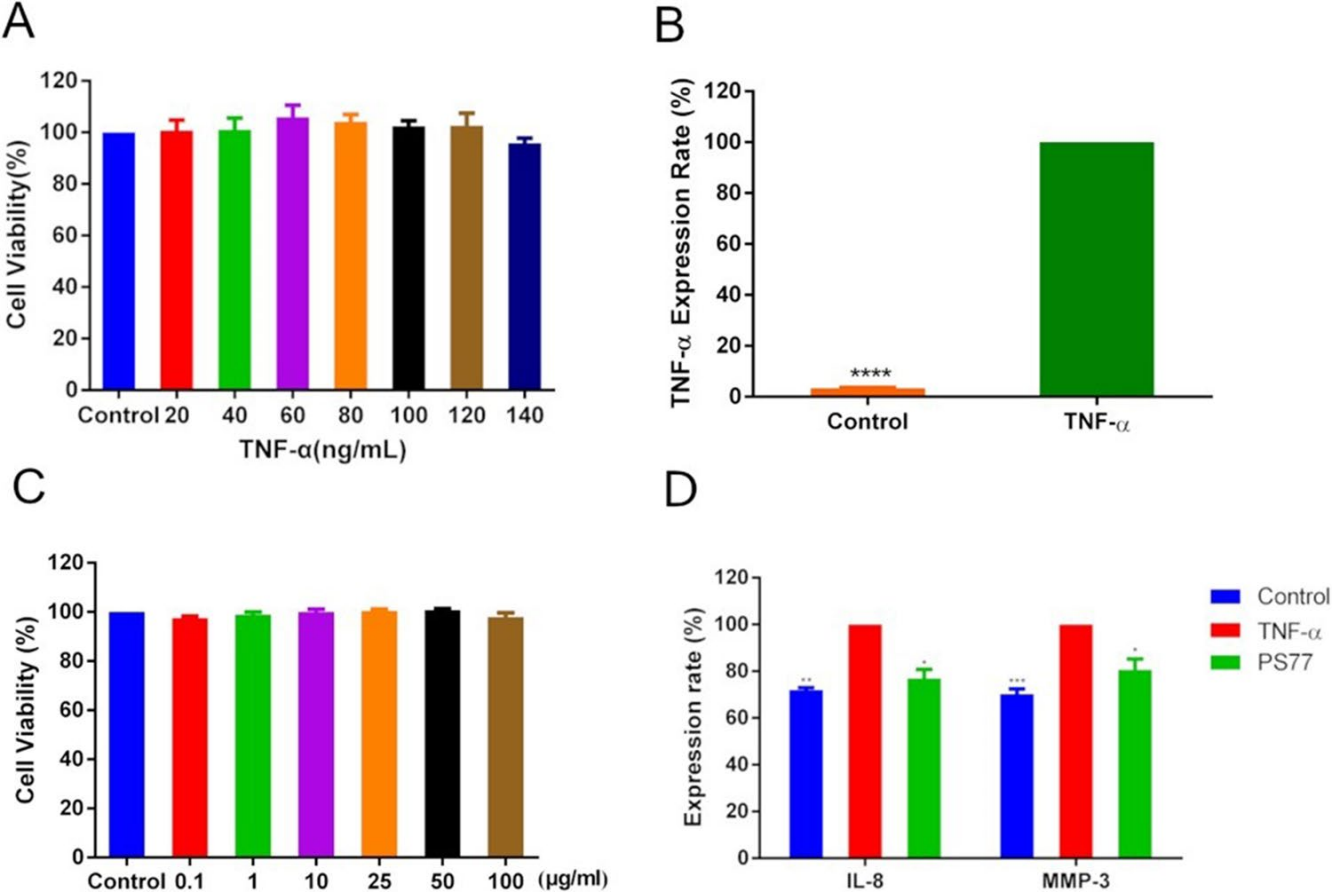
Effect and cytokine expression in the TNF-α-induced and PS77-treated on HaCaT cells.** A** Effect of TNF-α across different concentrations on normal HaCaT cells. **B** Cytokine expression in the TNF-α-induced inflammatory in model. **C** Effect of PS77 at different concentrations on normal HaCaT cells. **D** Cytokine expression in PS77-treated TNF-α-induced inflammatory model. The cell viability tests were averaged based on the cell viability of three independent biological replicates and statistically analyzed by Prism software, bar is shown as Mean ± SEM, *P < 0.05, **P < 0.01, ***P < 0.0005, ****P < 0.0001. TNF-α: TNF-α-induced inflammatory model, PS77: PS77-treated inflammatory model

To explore the effects of PS77 on inflammation, an in vitro model was established using human keratinocyte (HaCaT) cells treated with tumor necrosis factor-α (TNF-α). Cell viability assays showed no significant differences (*P* > 0.05) between normal HaCaT cells and those treated with TNF-α (20–140 ng/mL) for 2 h (Fig. 2A). TNF-α at 20 ng/mL was chosen for subsequent experiments. Enzyme-linked immunosorbent assay (ELISA) confirmed significantly increased (*P* < 0.05) TNF-α levels in the inflammatory model compared to normal controls (Fig. 2B), validating the model’s establishment.

### PS77 exhibits no significant toxicity on normal HaCaT cells

To explore the toxicity of PS77, cell viability assay was performed using normal HaCaT cells treated with the PS77 peptide (0.1–100 μg/mL) for 24 h. Our data showed that PS77-treated HaCaT cells in a concentration range of 0.1 to 100 μg/mL did not significantly affect the viability of normal HaCaT cells (cell viability was approximately 100%), suggesting its safety for potential therapeutic use (Fig. 2C). A concentration of 0.1 μg/mL was selected for further studies.

To examine the anti-inflammatory activity of PS77, we treated inflammatory model with 0.1 μg/mL of PS77 and examined the expression of the sets of inflammatory markers using ELISA assay. Our analysis revealed that PS77 treatment (0.1 μg/mL) significantly reduced (*P* < 0.05) the expression of pro-inflammatory cytokines IL-8 and MMP-3 by 20–30% in the TNF-α-induced inflammatory model, indicating PS77's anti-inflammatory effects (Fig. 2D). The pro-inflammatory cytokine expression rates were reduced 20 to 30% after the PS77 treatment, indicating that the anti-inflammatory effect of PS77 might be through the downregulation of the expression of these pro-inflammatory cytokines.

### Transcriptomic analysis of PS77-treated inflammatory model

To elucidate the mechanisms underlying PS77's anti-inflammatory activity, whole-transcriptome sequencing was performed on the PS77-treated inflammatory model (five biological replicates) and untreated controls. High-quality RNA samples (Supplementary Table 2) yielded 722.7 million paired-end reads (148 bp) with > 94% Q30 scores (Supplementary Table 3). Reads were mapped to the human reference genome using Hisat2(v2.0.1)1, with mapping rates > 92%, indicating the high quality of our transcriptomic data and is suitable for downstream analyses (Supplementary Table 4).

**Fig. 3:**
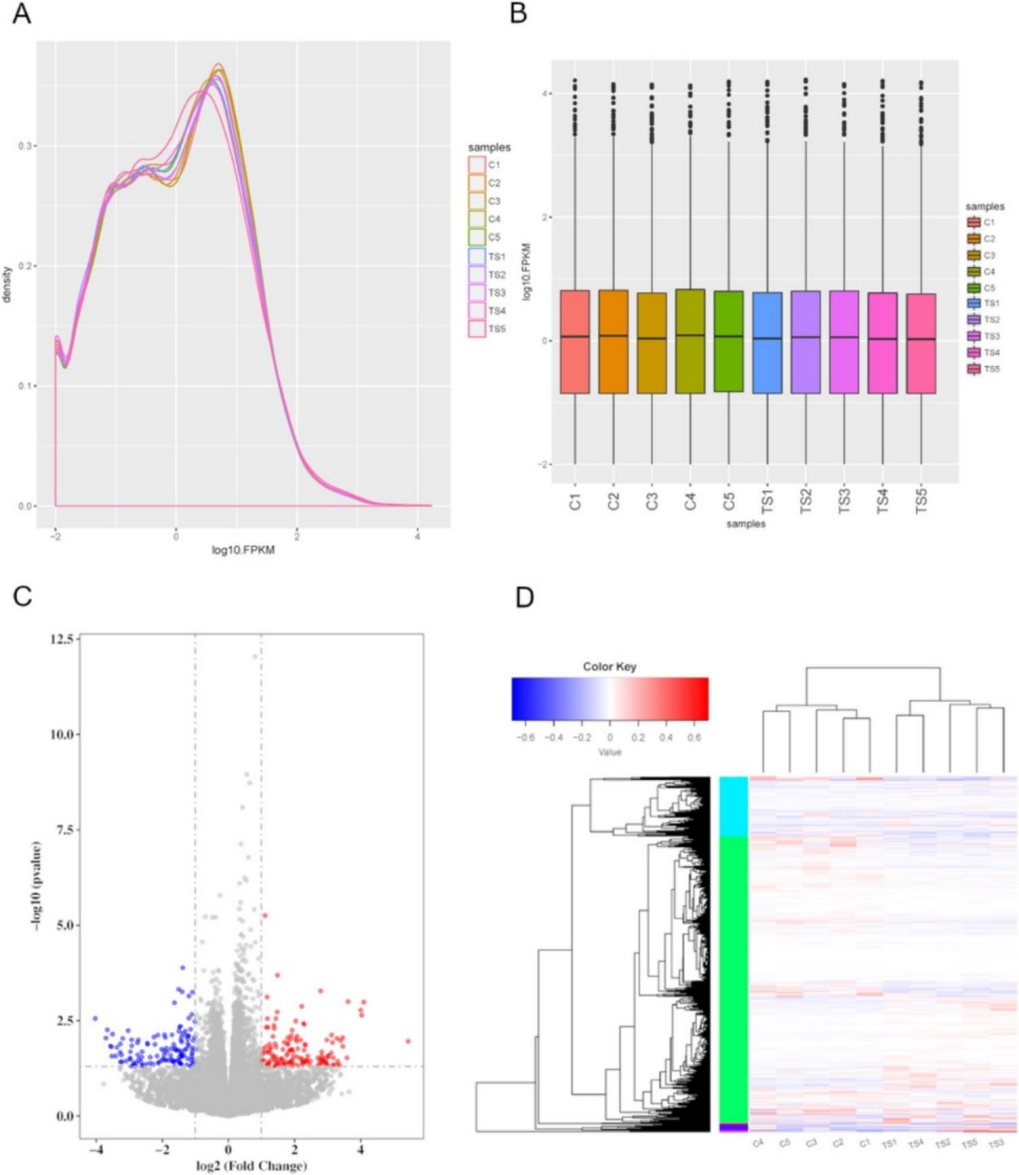
**A** FPKM density distribution correlation plots. **B** Box plots of gene expression level for each sample. **C** Volcano plot of DEGs regulated by PS77. **D** Heatmap of DEGs in different samples

Gene expression analysis revealed similar profiles between PS77-treated and control samples (Fig. 3A–B), suggesting few differentially expressed genes (DEGs) upon treatment. However, 265 significant DEGs (FC ≥ 2, *P* < 0.05) were identified, with 137 upregulated and 128 downregulated genes (Fig. 3C–D, Table 1 & Supplementary Table 5). Several of the downregulated genes are related to inflammatory and immune processes (Table 1). For instance, the key inflammation-related gene CHRNA7 is a cholinergic receptor that can influence inflammatory responses [27]. INHBB and CXCR5, cytokine and chemokine receptors involved in immune cell signaling and migration [28], were also downregulated. CAND2 and KLK4 are involved in cullin-associated protein degradation, which can modulate inflammatory signaling pathways. Notably, genes involved in the bone morphogenetic protein (BMP) signaling pathway, such as BMP5 and DLX5, were downregulated. The downregulation of these genes by PS77 aligns with its anti-inflammatory effects, as reducing the expression of pro-inflammatory genes would help dampen inflammation. Some upregulated genes also have potential anti-inflammatory roles. RXRG is a retinoid X receptor gamma, which can form complexes that inhibit inflammatory gene expression [29], whereas both IL12RB2 [30] and IL2RG are subunits of interleukin receptors that modulate interleukin signaling and can have immunoregulatory effects [31, 32], while anti-inflammatory genes.

**Table 1:** List of selected DEGs

Gene id	Fold Change	Gene Symbol	Regulation	Description
ENSG00000144712	16.3	CAND2	down	cullin associated and neddylation dissociated 2 (putative)
ENSG00000167749	9.42	KLK4	down	kallikrein related peptidase 4
ENSG00000105880	7.13	DLX5	down	distal-less homeobox 5
ENSG00000174123	6.59	TLR10	down	toll like receptor 10
ENSG00000112175	6.11	BMP5	down	bone morphogenetic protein 5
ENSG00000205809	5.43	KLRC2	down	killer cell lectin like receptor C2
ENSG00000164659	3.82	ELAPOR2	down	endosome-lysosome associated apoptosis and autophagy regulator family member 2
ENSG00000175344	3.17	CHRNA7	down	cholinergic receptor nicotinic alpha 7 subunit
ENSG00000136378	2.68	ADAMTS7	down	ADAM metallopeptidase with thrombospondin type 1 motif 7
ENSG00000163083	2.29	INHBB	down	inhibin subunit beta B
ENSG00000160683	2.21	CXCR5	down	C-X-C motif chemokine receptor 5
ENSG00000143171	17.08	RXRG	up	retinoid X receptor gamma
ENSG00000081985	8.85	IL12RB2	up	interleukin 12 receptor subunit beta 2
ENSG00000125618	7.07	PAX8	up	paired box 8
ENSG00000147168	4.53	IL2RG	up	interleukin 2 receptor subunit gamma

### Functional enrichment analysis of DEGs

**Fig. 4:**
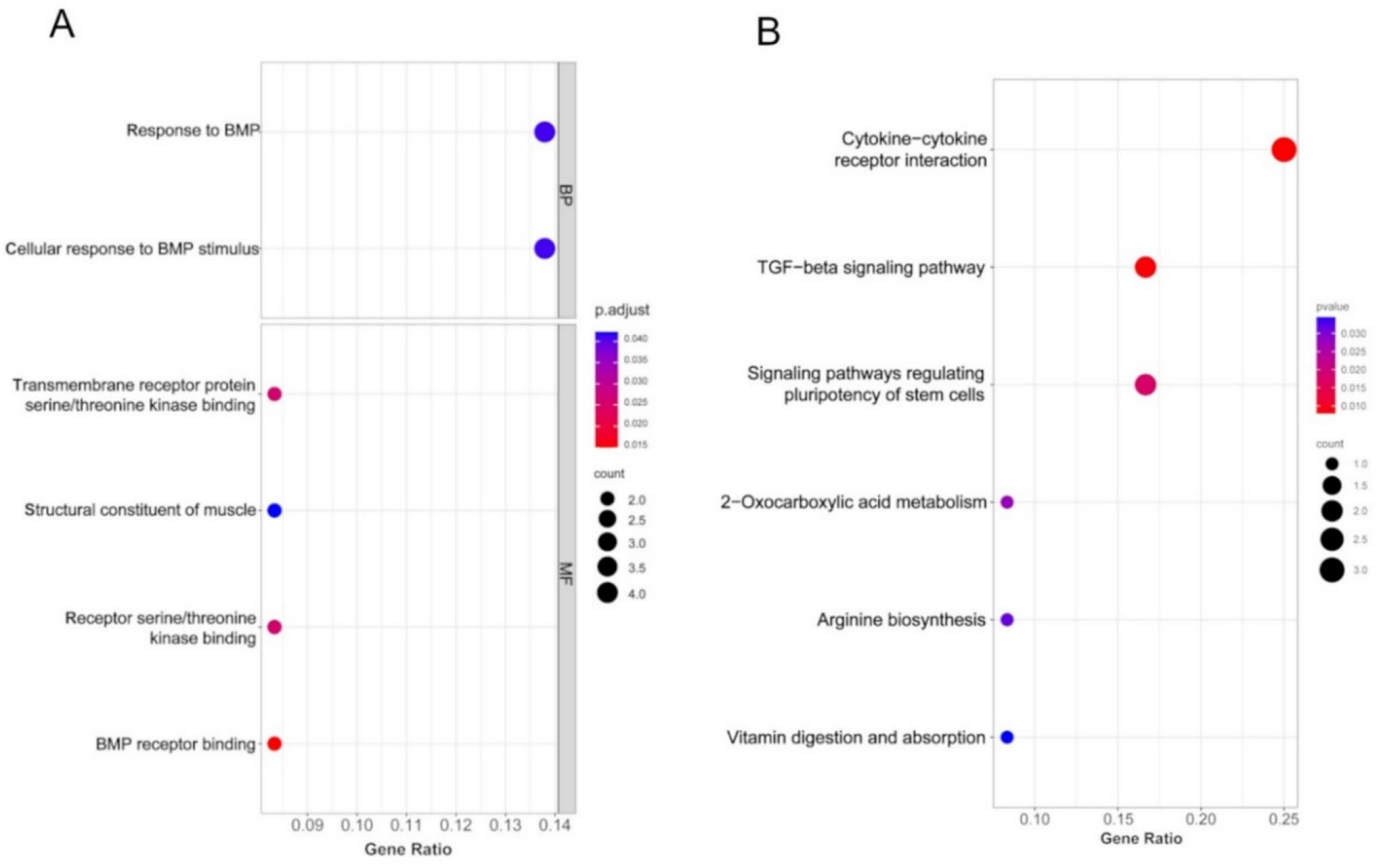
**A** GO biological process and molecular function enrichment analysis of all the downregulated genes. **B** KEGG pathway analysis of downregulated DEGs regulated by PS77

To have better insights into the functions of these downregulated DEGs, we performed the functional enrichment analysis using the provided tool on the Gene Ontology website (http://geneontology.org/). Our data revealed that downregulated DEGs were involved in biological processes such as response to BMP and cellular response to BMP stimulus, with genes including DLX5, BMP5, ELAPOR2, and ADAMTS7 (Fig. 4A). BMPs are members of the TGF-β superfamily and have been implicated in various inflammatory processes [33]. DLX5 has been reported to regulate chondrocyte hypertrophy [34], which is associated with osteoarthritis, a chronic inflammatory condition. By downregulating DLX5, PS77 may help to mitigate inflammation in osteoarthritis. However, the downregulation of genes involved in BMP signaling, such as DLX5 and BMP5, suggests that PS77 may exert its anti-inflammatory effects by modulating the BMP signaling pathway. Molecular functions enriched among downregulated DEGs included BMP receptor binding, transmembrane receptor protein serine/threonine kinase binding, receptor serine/threonine kinase binding, and structural constituent of muscle, with genes such as BMP5, ELAPOR2, MYH11, and SORBS2 (Fig. 4A).

### Pathway enrichment analysis

To understand and demonstrate the biochemical metabolic pathways and signal transduction pathways involved in DEGs, we performed pathway analysis using the provide tool on the KEGG [35]. Our analysis showed that the anti-inflammatory peptide PS77 downregulated genes that were enriched in several key pathways related to inflammation and cell signaling. The most significantly enriched pathway was the cytokine-cytokine receptor interaction, with a gene ratio of approximately 0.25. Cytokines are crucial mediators of inflammation, and their interaction with specific receptors triggers various signaling cascades that regulate the inflammatory response. The downregulation of genes involved in this pathway by PS77 suggests that the peptide may modulate the signaling of various cytokines, which are known to play crucial roles in inflammation [36]. By reducing the expression of these pro-inflammatory cytokines and their receptors, PS77 may help to dampen the inflammatory response. Another important pathway downregulated by PS77 was the TGF-beta signaling pathway, with a gene ratio of around 0.15 and involving genes such as BMP5, INHBB, and CXCR5 (Fig. 4B). The TGF-beta superfamily, which includes bone morphogenetic proteins (BMPs), plays a significant role in regulating inflammation, cell proliferation, and differentiation. INHBB, a member of the TGF-β superfamily, has been shown to promote the activation of fibroblasts and the production of pro-inflammatory cytokines [37]. By downregulating INHBB, PS77 may help to reduce inflammation and fibrosis. PS77 also downregulated genes involved in the 2-oxocarboxylic acid metabolism, arginine biosynthesis, and vitamin digestion and absorption pathways, although to a lesser extent (gene ratios < 0.10). For instance, arginine metabolism has been linked to the regulation of immune responses and inflammation [38]. By altering arginine biosynthesis, PS77 may influence the availability of this amino acid for immune cell function and cytokine production. These pathways are involved in various cellular processes, and their modulation by PS77 may indirectly contribute to its anti-inflammatory effects by altering cellular metabolism and nutrient availability.

Altogether our functional and pathway enrichment analyses provide valuable insights into the potential mechanisms underlying the anti-inflammatory effects of PS77. By modulating key inflammatory pathways, such as BMP and TGF-β signaling, and by regulating the expression of pro-inflammatory cytokines and their receptors, PS77 may exert a potent anti-inflammatory action. These findings support the further development of PS77 as a novel therapeutic agent for inflammatory diseases.

## Discussions

In this study, we discovered and synthesized a novel peptide, PS77, inspired by the TCM Squama Manitis, and investigated its anti-inflammatory properties using an in vitro TNF-α-induced inflammatory model in HaCaT cells. Our results demonstrate that PS77 effectively reduces the expression of pro-inflammatory cytokines, such as IL-8 and MMP-3, without exhibiting significant toxicity to normal human keratinocytes.

Transcriptomic analysis revealed that PS77 treatment led to the differential expression of 265 genes, with 137 upregulated and 128 downregulated. Among the downregulated genes, several key inflammation-related genes, such as CHRNA7 and CXCR5, were identified. CHRNA7 has been implicated in the regulation of inflammation in non-alcoholic fatty liver disease [39], while CXCR5 is known to play a role in cancer-related inflammation and immune responses [40]. The downregulation of these genes by PS77 suggests its potential to modulate inflammation through various signaling pathways, such as the BMP and TGF-β pathways.

Functional enrichment analysis of the downregulated genes revealed their involvement in biological processes related to the response to BMP and cellular response to BMP stimulus. BMPs are members of the TGF-β superfamily and have been shown to play a crucial role in regulating inflammation [41, 42]. The downregulation of BMP5 and other BMP-related genes, such as DLX5, ELAPOR2, and ADAMTS7, by PS77 indicates that it may exert its anti-inflammatory effects by modulating the BMP signaling pathway. In osteoarthritis, a chronic inflammatory disease affecting bone joints, BMP5 has been found to regulate chondrocyte growth through the ERK/p38 signaling pathway [43]. Downregulating BMP5 expression can inhibit the activation of p38 and ERK MAP kinases, reducing chondrocyte apoptosis and senescence, and ultimately mitigating the expression of inflammatory factors TNF-α and IL-62. Similarly, DLX5, a nuclear transcription factor, plays an important role in anti-inflammation by avoiding chondrocyte hypertrophy [34], which is associated with osteoarthritis [44]. Our data suggest that PS77 may be a potential therapeutic agent for osteoarthritis by downregulating BMP5 and DLX5, thereby suppressing the expression of pro-inflammatory cytokines.

The modulation of serine/threonine kinase (STK) binding activity by PS77 may also contribute to its anti-inflammatory effects, as STKs regulate IL-17, TNF, and IL-1β-induced inflammatory pathways [45]. Our data showed that BMP5 and ELAPOR2 could inhibit the binding of STK receptors, which are crucial for transducing signals from the extracellular to the intracellular space. PS77 may reduce the ability of STK binding by suppressing BMP5, leading to decreased production of cytokines.

Pathway enrichment analysis showed that the downregulated genes were enriched in cytokine-cytokine receptor interaction and TGF-β signaling pathways. By downregulating genes involved in these pathways, such as INHBB, PS77 may suppress the pro-inflammatory effects of TGF-β and BMP signaling. INHBB, a member of the TGF-β superfamily, is positively associated with the infiltration of macrophages, endothelial cells, and cancer-associated fibroblasts [46]. Inhibition of INHBB can block the activation of mesenchymal fibroblasts and ameliorate renal fibrosis [47], which can be promoted by prolonged inflammation. Thus, PS77 may exert its potential regulatory effect on inflammatory factors by inhibiting the expression of key genes such as INHBB in the signaling pathway, thereby mitigating pro-inflammatory responses and tissue fibrosis caused by TNF-α.

Additionally, PS77 upregulated the expression of genes with anti-inflammatory roles, such as RXRG, KRT76, IL12RB2, and COLEC11. RXRG can form heterodimers with PPAR, participating in the PPAR signaling pathway [48]. The upregulation of RXRG may lead to the activation of the PPAR signaling pathway, suppressing the NF-κB signaling pathway and the expression of pro-inflammatory factors [49, 50]. The increased expression of KRT76 might inhibit the increase in regulatory T cells and pro-inflammatory cytokines [51], while the upregulation of IL12RB2 suggests a potential modulatory effect in reducing the expression of the pro-inflammatory cytokine IL-17 [52]. Furthermore, the upregulation of COLEC11 may provide a new strategy for treating rheumatoid arthritis and other inflammatory diseases by promoting the expression of the anti-inflammatory factor IL-10 and suppressing the activation of antigen-presenting cells [53].

Finally, our results showed that the CXCR5 gene was significantly downregulated after PS77 treatment. CXCR5 is expressed on CD4 + and CD8 + T cells and is considered a defining marker of follicle-assisted T (Tfh) cells, which is an early marker during the ongoing immune response [54]. CXCR5 is the only receptor for CXCL13, and the CXCR5/CXCL13 axis plays a very important role in regulating immune response [54]. Tertiary lymphoid organs (TLO) mostly exist in inflammatory diseases [55], and CXCL13 induces the formation of TLO [56]. Without CXCR5, TLO cannot develop. Therefore, the anti-inflammatory activity of PS77 may be due to downregulating the expression of CXCR5, further limiting TLO development. For instance, CXCR5 and CXCL13 have been detected in various inflammatory diseases. In autoimmune myocarditis mice models, increased mRNA and protein expression of CXCR5 and CXCL13 were found in myocardial tissue [57]. Similarly, in lupus pneumonia, the concentration of CXCL13 in serum and the proportion of CXCR5 + B cells were significantly increased, with levels of CXCR5 and CXCL13 associated with disease occurrence and prognosis [58]. Our experiments showed that PS77 treatment significantly downregulated CXCR5 expression, potentially reducing CXCL13 activation and inhibiting its pro-inflammatory effects, suggesting a possible strategy for treating inflammatory diseases.

While our findings provide valuable insights into the anti-inflammatory mechanisms of PS77, further research is needed to fully elucidate its molecular targets and potential therapeutic applications. Future studies should focus on validating the identified target genes and pathways using in vitro and in vivo models of inflammation. Additionally, the safety and efficacy of PS77 should be evaluated in preclinical and clinical settings to assess its translational potential as a novel anti-inflammatory agent.

The discovery and characterization of PS77, a novel peptide derived from TCM, represents a significant advancement in the field of biomaterials for anti-inflammatory therapies. PS77’s α-helical structure enables precise interaction with biological systems, targeting the inflammation process at a molecular level through the modulation of the BMP and TGF-β signaling pathways. This specificity enhances its therapeutic potential while minimizing off-target effects, addressing a critical challenge in current anti-inflammatory therapies. The structural insights into PS77 also open avenues for bioengineering, allowing for modifications to tailor its properties for specific therapeutic contexts or integration into complex delivery systems. Our study highlights the untapped potential of peptides as biomaterials for anti-inflammatory therapies and the importance of interdisciplinary approaches combining traditional knowledge with modern scientific methods. PS77 paves the way for innovative therapies that could significantly impact the treatment of inflammatory diseases.

Future work should focus on addressing the limitations of this study. The absence of in vivo data restricts a comprehensive understanding of PS77’s efficacy, safety, biocompatibility, and translational potential, which are essential for its clinical application. Therefore, future studies will involve conducting in vivo experiments using animal models of chronic inflammation to evaluate these parameters. Additionally, the molecular mechanisms underlying PS77’s anti-inflammatory effects remain unclear, and advanced techniques, such as proteomic analyses and functional assays, are needed to identify the signaling pathways and downstream targets involved. These efforts will provide a deeper understanding of PS77’s therapeutic potential and pave the way for its clinical translation.

## Conclusion

In conclusion, our study unveils PS77 as a groundbreaking peptide with considerable promise for biomaterial-based anti-inflammatory therapies. Demonstrating a potent capacity to modulate key inflammatory pathways, notably the BMP and TGF-β signaling cascades, and to regulate the expression of critical inflammation-associated genes, PS77 sets a new benchmark in the field. Its unique α-helical structure underpins a suite of desirable properties, from enhanced biocompatibility and specificity to structural adaptability, which are fundamental for the next generation of therapeutic biomaterials. Future research should focus on detailed mechanistic studies of PS77's interaction with cellular receptors and signaling pathways, the exploration of its efficacy in in vivo models of inflammation, and the potential for developing delivery systems that enhance its therapeutic index. By bridging traditional medicinal wisdom with cutting-edge biomaterial science, PS77 emerges as a beacon for future innovations in biomedicine, marking a significant step forward in our quest for precision medicine solutions to inflammatory diseases.

## Supplementary Information

Below is the link to the electronic supplementary material.

Supplementary file1 (DOCX 60.2 KB)

## Data Availability

Data supporting the findings of this study are included in the published paper and additional information. The raw RNA-Seq sequence data was shared in the CNGB Sequence Archive (CNSA), please refer to the accession number: CNP0005582.
