# Supplementary material for: PS77: a novel peptide with α-helical structure for targeted anti-inflammatory therapy in biomaterials design
Source: Immunol Res. 2025 Jul 24;73(1):110. doi: 10.1007/s12026-025-09663-0 (PMC12289727; doi:10.1007/s12026-025-09663-0)
Supplement: Supplementary file 1 — Supplementary file1 (DOCX 60.2 KB) [file 12026_2025_9663_MOESM1_ESM.docx]

**Supplementary Table 1. The properties of the synthetic peptide PS77.**

| **Sequence** | **Molecular**  **Weight** | **HPLC**  **Purity**  **(%)** | **Appearance** | **Peptide Length**  **(AA)** |
| --- | --- | --- | --- | --- |
| RVFQHLVNLEVLR | 1622.92 | 95.3 | White lyophilized powder | 13 |

**Supplementary Table 2. RNA integrity assessment. C: Control group, TNF-α-induced inflammatory model.** TS: Treatment group, the PS77-treated TNF-α-induced inflammatory model.

| **Sample Name** | **Volume (μL)** | **RNA Concentration (ng/μL)** | **RNA Quality (μg)** | **A260/280** | **A260/230** | **RIN** |
| --- | --- | --- | --- | --- | --- | --- |
| C1 | 25 | 964.8 | 24.12 | 2.07 | 1.94 | 10 |
| C2 | 25 | 891.4 | 22.28 | 2.06 | 1.62 | 10 |
| C3 | 25 | 969.4 | 24.24 | 2.08 | 1.81 | 10 |
| C4 | 25 | 902.3 | 22.56 | 2.06 | 1.27 | 10 |
| C5 | 25 | 941.7 | 23.54 | 2.07 | 2.13 | 10 |
| TS1 | 25 | 1062.2 | 26.56 | 2.06 | 2.03 | 10 |
| TS2 | 25 | 1016.7 | 25.42 | 2.04 | 1.5 | 10 |
| TS3 | 25 | 933.2 | 23.33 | 2.04 | 2.08 | 10 |
| TS4 | 25 | 851.9 | 21.3 | 2.04 | 2.04 | 10 |
| TS5 | 25 | 903.2 | 22.58 | 2.04 | 2.12 | 10 |

**Supplementary Table 3. Summary statistics of sequencing clean reads.**

| **Sample Name** | **Length** | **Number of Reads** | **Number of Base** | **Q20 (%)** | **Q30 (%)** | **GC (%)** | **N (ppm)** |
| --- | --- | --- | --- | --- | --- | --- | --- |
| C1 | 148.6 | 70955074 | 10545715516 | 98.15 | 94.84 | 51.75 | 6.5 |
| C2 | 148.8 | 70702538 | 10520946223 | 98.32 | 95.29 | 51.69 | 6.11 |
| C2 | 148.6 | 71640014 | 10644717610 | 98.16 | 94.92 | 52.59 | 6.04 |
| C1 | 148.4 | 75973278 | 11274856953 | 98.14 | 94.85 | 51.93 | 6.99 |
| C2 | 148.2 | 68612642 | 10168248966 | 97.91 | 94.33 | 52.45 | 6.99 |
| TS1 | 148.5 | 77268128 | 11472506307 | 98.19 | 94.88 | 52.69 | 0.59 |
| TS2 | 148.4 | 69847690 | 10367981543 | 98.18 | 94.95 | 52.17 | 6.32 |
| TS3 | 148.5 | 76547106 | 11366216910 | 98.13 | 94.79 | 52.32 | 6.97 |
| TS4 | 148.4 | 70962906 | 10529357136 | 98.25 | 95.07 | 52.45 | 6.94 |
| TS5 | 148.4 | 70211470 | 10418952621 | 98.21 | 95.04 | 52.79 | 6.53 |

**Supplementary Table 4. Mapping statistics of clean reads.**

| **Sample**  **Name** | **Total Reads** | **Total**  **Mapped (%)** | **Multiple**  **Mapped (%)** | **Uniquely**  **Mapped (%)** | **Read 1** | **Read 2** |
| --- | --- | --- | --- | --- | --- | --- |
| C1 | 70955074 | 94.2 | 7.8 | 86.4 | 30822418 | 30496293 |
| C2 | 70702538 | 94.4 | 7.8 | 86.6 | 30751869 | 30479076 |
| C3 | 71640014 | 93.4 | 8.3 | 85.1 | 30674982 | 30297515 |
| C4 | 75973278 | 94.0 | 7.6 | 86.3 | 32958519 | 32618804 |
| C5 | 68612642 | 93.3 | 8.0 | 85.3 | 29502871 | 29032861 |
| TS1 | 77268128 | 93.1 | 8.1 | 84.9 | 32980829 | 32639550 |
| TS2 | 69847690 | 94.6 | 7.8 | 86.8 | 30478278 | 30150400 |
| TS3 | 76547106 | 92.8 | 8.0 | 84.8 | 32638108 | 32293918 |
| TS4 | 70962906 | 95.2 | 8.2 | 87.0 | 30984928 | 30726804 |
| TS5 | 70211470 | 92.9 | 8.6 | 84.3 | 29727820 | 29462218 |

# **Supplementary Table 5. List of up regulated DGEs of PS77 treatment.**

| gene_Id | Fold Change | Gene Symbol | Regulation | Description |
| --- | --- | --- | --- | --- |
| ENSG00000163395 | 43.38 | IGFN1 | up | immunoglobulin like and fibronectin type III domain containing 1 |
| ENSG00000143171 | 17.08 | RXRG | up | retinoid X receptor gamma |
| ENSG00000230648 | 16.34 | - | up | novel transcript |
| ENSG00000079931 | 16.04 | MOXD1 | up | monooxygenase DBH like 1 |
| ENSG00000270104 | 12.30 | - | up | novel transcript |
| ENSG00000183148 | 12.00 | ANKRD20A2P | up | ankyrin repeat domain 20 family member A2%2C pseudogene |
| ENSG00000156510 | 11.03 | HKDC1 | up | hexokinase domain containing 1 |
| ENSG00000185069 | 11.01 | KRT76 | up | keratin 76 |
| ENSG00000244094 | 10.50 | SPRR2F | up | small proline rich protein 2F |
| ENSG00000236444 | 10.26 | UBE2L5 | up | ubiquitin conjugating enzyme E2 L5 |
| ENSG00000259351 | 9.87 | - | up | novel transcript%2C antisense to DAPK2 |
| ENSG00000237766 | 9.71 | GGTA2P | up | glycoprotein alpha-galactosyltransferase 2%2C pseudogene |
| ENSG00000235672 | 9.68 | - | up | RAN binding protein 1 (RANBP1) pseudogene |
| ENSG00000205863 | 9.02 | C1QTNF9B | up | C1q and TNF related 9B |
| ENSG00000204663 | 9.02 | CST13P | up | cystatin 13%2C pseudogene |
| ENSG00000275630 | 8.97 | - | up | novel transcript |
| ENSG00000081985 | 8.85 | IL12RB2 | up | interleukin 12 receptor subunit beta 2 |
| ENSG00000280331 | 8.71 | - | up | TEC |
| ENSG00000197479 | 8.64 | PCDHB11 | up | protocadherin beta 11 |
| ENSG00000173110 | 8.13 | HSPA6 | up | heat shock protein family A (Hsp70) member 6 |
| ENSG00000206630 | 8.10 | SNORD60 | up | small nucleolar RNA%2C C/D box 60 |
| ENSG00000249715 | 7.99 | FER1L5 | up | fer-1 like family member 5 |
| ENSG00000270141 | 7.91 | TERC | up | telomerase RNA component |
| ENSG00000242516 | 7.88 | LINC00960 | up | long intergenic non-protein coding RNA 960 |
| ENSG00000225727 | 7.76 | - | up | filamin A interacting protein 1-like (FILIP1L) pseudogene |
| ENSG00000188460 | 7.63 | ACTBP11 | up | ACTB pseudogene 11 |
| ENSG00000154654 | 7.57 | NCAM2 | up | neural cell adhesion molecule 2 |
| ENSG00000232132 | 7.30 | NDFIP2-AS1 | up | NDFIP2 antisense RNA 1 |
| ENSG00000114279 | 7.24 | FGF12 | up | fibroblast growth factor 12 |
| ENSG00000262362 | 7.16 | - | up | novel transcript%2C sense overlapping CLDN6 |
| ENSG00000255750 | 7.09 | - | up | novel transcript%2C antisense to SSPN |
| ENSG00000125618 | 7.07 | PAX8 | up | paired box 8 |
| ENSG00000161992 | 7.01 | PRR35 | up | proline rich 35 |
| ENSG00000175426 | 6.91 | PCSK1 | up | proprotein convertase subtilisin/kexin type 1 |
| ENSG00000259038 | 6.76 | - | up | novel transcript%2C antisense to RAD51L1 |
| ENSG00000118004 | 6.74 | COLEC11 | up | collectin subfamily member 11 |
| ENSG00000285467 | 6.60 | - | up | novel transcript |
| ENSG00000278048 | 6.55 | U2 | up | U2 spliceosomal RNA |
| ENSG00000253485 | 5.59 | PCDHGA5 | up | protocadherin gamma subfamily A%2C 5 |
| ENSG00000205054 | 5.47 | LINC01121 | up | long intergenic non-protein coding RNA 1121 |
| ENSG00000171711 | 5.28 | DEFB4A | up | defensin beta 4A |
| ENSG00000004468 | 5.18 | CD38 | up | CD38 molecule |
| ENSG00000196593 | 5.15 | ANKRD20A19P | up | ankyrin repeat domain 20 family member A19%2C pseudogene |
| ENSG00000228237 | 5.07 | EFCAB14-AS1 | up | EFCAB14 antisense RNA 1 |
| ENSG00000224261 | 5.01 | RPSAP18 | up | ribosomal protein SA pseudogene 18 |
| ENSG00000258303 | 4.99 | - | up | novel transcript |
| ENSG00000260342 | 4.90 | - | up | novel protein |
| ENSG00000267370 | 4.89 | - | up | zinc finger protein 420 (ZNF420) pseudogene |
| ENSG00000286445 | 4.83 | - | up | novel transcript |
| ENSG00000236526 | 4.83 | - | up | novel transcript |
| ENSG00000258791 | 4.73 | LINC00520 | up | long intergenic non-protein coding RNA 520 |
| ENSG00000183607 | 4.70 | GKN2 | up | gastrokine 2 |
| ENSG00000204174 | 4.69 | NPY4R | up | neuropeptide Y receptor Y4 |
| ENSG00000169474 | 4.66 | SPRR1A | up | small proline rich protein 1A |
| ENSG00000147168 | 4.53 | IL2RG | up | interleukin 2 receptor subunit gamma |
| ENSG00000283189 | 4.53 | - | up | novel protein |
| ENSG00000276255 | 4.29 | LINC02809 | up | long intergenic non-protein coding RNA 2809 |
| ENSG00000257515 | 4.28 | - | up | novel transcript |
| ENSG00000188375 | 4.21 | H3-5 | up | H3.5 histone |
| ENSG00000249846 | 4.20 | LINC02021 | up | long intergenic non-protein coding RNA 2021 |
| ENSG00000073146 | 4.18 | MOV10L1 | up | Mov10 like RISC complex RNA helicase 1 |
| ENSG00000235508 | 4.11 | RPS2P7 | up | ribosomal protein S2 pseudogene 7 |
| ENSG00000228140 | 4.09 | - | up | novel transcript |
| ENSG00000226862 | 4.03 | - | up | novel transcript |
| ENSG00000268583 | 3.89 | - | up | novel transcript%2C antisense to CARD8 |
| ENSG00000171126 | 3.88 | KCNG3 | up | potassium voltage-gated channel modifier subfamily G member 3 |
| ENSG00000235180 | 3.84 | LINC00601 | up | long intergenic non-protein coding RNA 601 |
| ENSG00000287929 | 3.82 | - | up | novel transcript%2C antisense to NMNAT2 |
| ENSG00000262768 | 3.81 | - | up | novel transcript%2C antisense to CBX8 |
| ENSG00000225693 | 3.79 | LAGE3P1 | up | L antigen family member 3 pseudogene 1 |
| ENSG00000226571 | 3.75 | - | up | novel transcript |
| ENSG00000147872 | 3.73 | PLIN2 | up | perilipin 2 |
| ENSG00000177770 | 3.72 | CDKN2AIPNLP1 | up | CDKN2A interacting protein N-terminal like pseudogene 1 |
| ENSG00000205670 | 3.71 | SMIM11A | up | small integral membrane protein 11A |
| ENSG00000254595 | 3.68 | - | up | voltage-dependent anion channel 1(VDAC1) pseudogene |
| ENSG00000227582 | 3.66 | ADGRF5P1 | up | adhesion G protein-coupled receptor F5 pseudogene 1 |
| ENSG00000287851 | 3.63 | - | up | novel transcript |
| ENSG00000213598 | 3.60 | - | up | ribosomal protein L31 (RPL31) pseudogene |
| ENSG00000272582 | 3.49 | - | up | novel transcript%2C antisense to C22orf23 |
| ENSG00000276571 | 3.48 | - | up | novel transcript%2C antisense to C16orf62 |
| ENSG00000267472 | 3.48 | ARHGAP27P2 | up | Rho GTPase activating protein 27 pseudogene 2 |
| ENSG00000259015 | 3.47 | - | up | chromosome 17 open reading frame 98 (C17orf98) pseudogene |
| ENSG00000274929 | 3.47 | - | up | novel transcript%2C sense intronic to RCBTB2 |
| ENSG00000234036 | 3.40 | TXNP6 | up | thioredoxin pseudogene 6 |
| ENSG00000272701 | 3.30 | MESTIT1 | up | MEST intronic transcript 1%2C antisense RNA |
| ENSG00000259052 | 3.24 | - | up | novel transcript%2C sense overlapping to SLC25A29 |
| ENSG00000250007 | 3.22 | - | up | novel transcript%2C antisense to ACTC1 |
| ENSG00000158683 | 3.22 | PKD1L1 | up | polycystin 1 like 1%2C transient receptor potential channel interacting |
| ENSG00000284237 | 3.21 | LINC02767 | up | long intergenic non-protein coding RNA 2767 |
| ENSG00000100985 | 3.14 | MMP9 | up | matrix metallopeptidase 9 |
| ENSG00000268707 | 3.11 | - | up | novel transcript |
| ENSG00000267119 | 3.04 | RPL10P15 | up | ribosomal protein L10 pseudogene 15 |
| ENSG00000213542 | 3.04 | - | up | ribosomal protein L7a (RPL7A) pseudogene |
| ENSG00000283900 | 2.95 | TPTEP2-CSNK1E | up | TPTEP2-CSNK1E readthrough |
| ENSG00000238121 | 2.87 | LINC00426 | up | long intergenic non-protein coding RNA 426 |
| ENSG00000237039 | 2.83 | RPS28P4 | up | ribosomal protein S28 pseudogene 4 |
| ENSG00000241889 | 2.82 | - | up | dullard homolog (Xenopus laevis) (DULLARD) pseudogene |
| ENSG00000140465 | 2.80 | CYP1A1 | up | cytochrome P450 family 1 subfamily A member 1 |
| ENSG00000163485 | 2.78 | ADORA1 | up | adenosine A1 receptor |
| ENSG00000261783 | 2.73 | - | up | novel transcript%2C sense intronic to CFDP1 |
| ENSG00000087076 | 2.73 | HSD17B14 | up | hydroxysteroid 17-beta dehydrogenase 14 |
| ENSG00000114656 | 2.70 | CFAP92 | up | cilia and flagella associated protein 92 (putative) |
| ENSG00000074660 | 2.70 | SCARF1 | up | scavenger receptor class F member 1 |
| ENSG00000128274 | 2.67 | A4GALT | up | alpha 1%2C4-galactosyltransferase (P blood group) |
| ENSG00000205754 | 2.66 | SLCO1B7 | up | solute carrier organic anion transporter family member 1B7 (putative) |
| ENSG00000230882 | 2.66 | - | up | hypothetical protein LOC285908 (LOC285908) pseudogene |
| ENSG00000275426 | 2.63 | - | up | novel transcript |
| ENSG00000179165 | 2.61 | PXT1 | up | peroxisomal testis enriched protein 1 |
| ENSG00000125510 | 2.60 | OPRL1 | up | opioid related nociceptin receptor 1 |
| ENSG00000153093 | 2.55 | ACOXL | up | acyl-CoA oxidase like |
| ENSG00000176919 | 2.55 | C8G | up | complement C8 gamma chain |
| ENSG00000273703 | 2.53 | H2BC14 | up | H2B clustered histone 14 |
| ENSG00000279458 | 2.49 | - | up | novel transcript |
| ENSG00000124159 | 2.45 | MATN4 | up | matrilin 4 |
| ENSG00000170927 | 2.39 | PKHD1 | up | PKHD1 ciliary IPT domain containing fibrocystin/polyductin |
| ENSG00000118523 | 2.38 | CCN2 | up | cellular communication network factor 2 |
| ENSG00000103522 | 2.37 | IL21R | up | interleukin 21 receptor |
| ENSG00000105967 | 2.33 | TFEC | up | transcription factor EC |
| ENSG00000256268 | 2.33 | LINC02454 | up | long intergenic non-protein coding RNA 2454 |
| ENSG00000287910 | 2.33 | - | up | novel transcript |
| ENSG00000268173 | 2.29 | - | up | novel protein%2C readthrough between PIK3R2 and IFI30 |
| ENSG00000278899 | 2.29 | - | up | novel transcript%2C antisense to LATS1 |
| ENSG00000266964 | 2.28 | FXYD1 | up | FXYD domain containing ion transport regulator 1 |
| ENSG00000234699 | 2.26 | - | up | novel transcript |
| ENSG00000179698 | 2.25 | WDR97 | up | WD repeat domain 97 |
| ENSG00000178150 | 2.25 | ZNF114 | up | zinc finger protein 114 |
| ENSG00000277501 | 2.22 | - | up | novel transcript%2C antisense DDX52 |
| ENSG00000186326 | 2.21 | RGS9BP | up | regulator of G protein signaling 9 binding protein |
| ENSG00000287539 | 2.20 | - | up | novel transcript |
| ENSG00000204421 | 2.19 | LY6G6C | up | lymphocyte antigen 6 family member G6C |
| ENSG00000187912 | 2.16 | CLEC17A | up | C-type lectin domain containing 17A |
| ENSG00000150275 | 2.11 | PCDH15 | up | protocadherin related 15 |
| ENSG00000279504 | 2.05 | - | up | TEC |
| ENSG00000162949 | 2.04 | CAPN13 | up | calpain 13 |
| ENSG00000246203 | 2.03 | - | up | novel pseudogene |
| ENSG00000258325 | 2.02 | ITFG2-AS1 | up | ITFG2 antisense RNA 1 |
| ENSG00000286048 | 2.00 | - | up | novel transcript%2C antisense to ITGA2 |

# Supplementary Table 6 - List of down regulated DGEs of PS77 treatment.

| Gene Id | Fold Change | Gene Symbol | Regulation | Description |
| --- | --- | --- | --- | --- |
| ENSG00000144712 | 16.30 | CAND2 | down | cullin associated and neddylation dissociated 2 (putative) |
| ENSG00000260641 | 13.03 | - | down | novel transcript%2C antisense TSPAN5 |
| ENSG00000237301 | 12.64 | - | down | novel transcript |
| ENSG00000271964 | 11.85 | - | down | novel transcript%2C antisense to RFTN1 |
| ENSG00000266446 | 11.62 | - | down | novel transcript%2C sense intronic CDKN2B-AS1 |
| ENSG00000251129 | 11.51 | LINC02506 | down | long intergenic non-protein coding RNA 2506 |
| ENSG00000271327 | 11.35 | - | down | novel transcript |
| ENSG00000286825 | 10.59 | - | down | novel transcript%2C antisense to RABGGTA |
| ENSG00000207721 | 10.35 | MIR186 | down | microRNA 186 |
| ENSG00000286757 | 9.67 | - | down | novel transcript%2C antisense to CLYBL |
| ENSG00000225891 | 9.62 | DHDDS-AS1 | down | DHDDS antisense RNA 1 |
| ENSG00000249388 | 9.58 | - | down | novel transcript |
| ENSG00000167749 | 9.42 | KLK4 | down | kallikrein related peptidase 4 |
| ENSG00000265490 | 9.34 | - | down | novel transcript%2C antisense to ENOSF1 |
| ENSG00000231752 | 9.17 | EMBP1 | down | embigin pseudogene 1 |
| ENSG00000273024 | 8.51 | INTS4P2 | down | integrator complex subunit 4 pseudogene 2 |
| ENSG00000260953 | 8.19 | - | down | novel transcript |
| ENSG00000272911 | 7.86 | - | down | novel transcript |
| ENSG00000171786 | 7.80 | NHLH1 | down | nescient helix-loop-helix 1 |
| ENSG00000276115 | 7.80 | - | down | novel transcript%2C sense intronic to BICD1 |
| ENSG00000287582 | 7.65 | - | down | novel transcript |
| ENSG00000242444 | 7.61 | - | down | ribosomal protein L18 (RPL18) pseudogene |
| ENSG00000003987 | 7.59 | MTMR7 | down | myotubularin related protein 7 |
| ENSG00000258922 | 7.28 | - | down | novel transcript |
| ENSG00000233122 | 7.28 | CTAGE7P | down | CTAGE family member 7%2C pseudogene |
| ENSG00000105880 | 7.13 | DLX5 | down | distal-less homeobox 5 |
| ENSG00000233739 | 7.04 | - | down | novel transcript |
| ENSG00000267699 | 6.89 | - | down | novel protein |
| ENSG00000256973 | 6.72 | - | down | novel transcript |
| ENSG00000244009 | 6.68 | B3GAT3P1 | down | beta-1%2C3-glucuronyltransferase 3 pseudogene 1 |
| ENSG00000255647 | 6.61 | - | down | novel transcript |
| ENSG00000174123 | 6.59 | TLR10 | down | toll like receptor 10 |
| ENSG00000114786 | 6.29 | ABHD14A-ACY1 | down | ABHD14A-ACY1 readthrough |
| ENSG00000238133 | 6.15 | MAP3K20-AS1 | down | MAP3K20 antisense RNA 1 |
| ENSG00000112175 | 6.11 | BMP5 | down | bone morphogenetic protein 5 |
| ENSG00000086205 | 6.08 | FOLH1 | down | folate hydrolase 1 |
| ENSG00000244125 | 6.08 | - | down | novel transcript%2C sense overlapping to LRP1B |
| ENSG00000260017 | 5.68 | - | down | novel transcript%2C antisense to SMG1 |
| ENSG00000089558 | 5.47 | KCNH4 | down | potassium voltage-gated channel subfamily H member 4 |
| ENSG00000205809 | 5.43 | KLRC2 | down | killer cell lectin like receptor C2 |
| ENSG00000221340 | 5.20 | RNU6ATAC18P | down | RNA%2C U6atac small nuclear 18%2C pseudogene |
| ENSG00000281538 | 5.15 | - | down | novel transcript |
| ENSG00000165164 | 4.86 | CFAP47 | down | cilia and flagella associated protein 47 |
| ENSG00000231327 | 4.77 | LINC01816 | down | long intergenic non-protein coding RNA 1816 |
| ENSG00000242337 | 4.67 | INHCAP | down | inhibitor of carbonic anhydrase pseudogene |
| ENSG00000272293 | 4.46 | - | down | novel transcript%2C antisense to FBXO25 |
| ENSG00000260743 | 4.39 | - | down | novel transcript |
| ENSG00000275329 | 4.36 | - | down | novel transcript |
| ENSG00000266171 | 4.28 | - | down | novel transcript%2C antisense to YES1 |
| ENSG00000258136 | 4.04 | - | down | novel transcript%2C antisense to PRDM4 |
| ENSG00000273374 | 4.04 | - | down | novel transcript |
| ENSG00000197927 | 3.92 | NBEAP2 | down | neurobeachin pseudogene 2 |
| ENSG00000279267 | 3.85 | - | down | TEC |
| ENSG00000281383 | 3.84 | - | down | novel transcript%2C similar to YY1 associated myogenesis RNA 1 YAM1 |
| ENSG00000164659 | 3.82 | ELAPOR2 | down | endosome-lysosome associated apoptosis and autophagy regulator family member 2 |
| ENSG00000224944 | 3.78 | CASC6 | down | cancer susceptibility 6 |
| ENSG00000272989 | 3.75 | LINC02012 | down | long intergenic non-protein coding RNA 2012 |
| ENSG00000171291 | 3.68 | ZNF439 | down | zinc finger protein 439 |
| ENSG00000224220 | 3.66 | DTNB-AS1 | down | DTNB antisense RNA 1 |
| ENSG00000186714 | 3.62 | CCDC73 | down | coiled-coil domain containing 73 |
| ENSG00000259607 | 3.44 | - | down | novel transcript%2C antisense to KIF13B |
| ENSG00000277310 | 3.35 | - | down | novel transcript%2C sense intronic to CCDC11 |
| ENSG00000287188 | 3.32 | - | down | novel transcript%2C antisense to ANXA10 |
| ENSG00000273448 | 3.26 | - | down | novel transcript |
| ENSG00000269397 | 3.26 | - | down | novel transcript%2C sense intronic to ZNF726 |
| ENSG00000265452 | 3.22 | MIR3682 | down | microRNA 3682 |
| ENSG00000175344 | 3.17 | CHRNA7 | down | cholinergic receptor nicotinic alpha 7 subunit |
| ENSG00000277423 | 3.15 | - | down | novel transcript |
| ENSG00000101342 | 3.10 | TLDC2 | down | TBC/LysM-associated domain containing 2 |
| ENSG00000223764 | 3.09 | LINC02593 | down | long intergenic non-protein coding RNA 2593 |
| ENSG00000248968 | 3.08 | - | down | novel transcript |
| ENSG00000248206 | 3.06 | - | down | novel transcript |
| ENSG00000203876 | 3.05 | ADD3-AS1 | down | ADD3 antisense RNA 1 |
| ENSG00000255811 | 2.94 | - | down | novel transcript%2C antisense to GJB4 and GJB3 |
| ENSG00000176771 | 2.89 | NCKAP5 | down | NCK associated protein 5 |
| ENSG00000161664 | 2.84 | ASB16 | down | ankyrin repeat and SOCS box containing 16 |
| ENSG00000165323 | 2.84 | FAT3 | down | FAT atypical cadherin 3 |
| ENSG00000267416 | 2.83 | HEATR6-DT | down | HEATR6 divergent transcript |
| ENSG00000265415 | 2.81 | - | down | novel transcript%2C antisense to PRR11 |
| ENSG00000261773 | 2.80 | - | down | novel transcript%2C overlapping to FAM3A |
| ENSG00000215115 | 2.78 | CXorf49 | down | chromosome X open reading frame 49 |
| ENSG00000230408 | 2.78 | BZW1-AS1 | down | BZW1 antisense RNA 1 |
| ENSG00000257653 | 2.74 | - | down | novel transcript%2C antisense to ADCY6 |
| ENSG00000273747 | 2.73 | - | down | novel transcript%2C antisense to BTBD1 |
| ENSG00000105538 | 2.71 | RASIP1 | down | Ras interacting protein 1 |
| ENSG00000279662 | 2.71 | - | down | TEC |
| ENSG00000203469 | 2.70 | - | down | novel transcript%2C antisense to DFFA |
| ENSG00000259327 | 2.69 | - | down | novel transcript |
| ENSG00000285667 | 2.69 | - | down | novel transcript |
| ENSG00000136378 | 2.68 | ADAMTS7 | down | ADAM metallopeptidase with thrombospondin type 1 motif 7 |
| ENSG00000133392 | 2.67 | MYH11 | down | myosin heavy chain 11 |
| ENSG00000257550 | 2.67 | - | down | novel transcript%2C antisense to ATF7 |
| ENSG00000131183 | 2.66 | SLC34A1 | down | solute carrier family 34 member 1 |
| ENSG00000284879 | 2.64 | - | down | novel transcript |
| ENSG00000266302 | 2.63 | - | down | novel transcript |
| ENSG00000285728 | 2.60 | - | down | novel transcript%2C antisense to PPIH |
| ENSG00000257222 | 2.59 | - | down | novel transcript%2C antisense to WASHC3 and NUP37 |
| ENSG00000168852 | 2.55 | TPTE2P5 | down | transmembrane phosphoinositide 3-phosphatase and tensin homolog 2 pseudogene 5 |
| ENSG00000278621 | 2.49 | THBS1-AS1 | down | THBS1 antisense RNA 1 |
| ENSG00000127831 | 2.46 | VIL1 | down | villin 1 |
| ENSG00000246214 | 2.42 | - | down | novel transcript%2C antisense to FAM134B |
| ENSG00000273568 | 2.41 | - | down | novel transcript%2C sense intronic to PUS1 |
| ENSG00000238363 | 2.40 | SNORA13 | down | small nucleolar RNA%2C H/ACA box 13 |
| ENSG00000254835 | 2.36 | RNF185-AS1 | down | RNF185 antisense RNA 1 |
| ENSG00000249379 | 2.36 | - | down | novel transcript |
| ENSG00000262211 | 2.34 | - | down | novel transcript%2C antisense to IL6ST |
| ENSG00000272056 | 2.31 | - | down | novel transcript%2C antisense to AGBL5 |
| ENSG00000188933 | 2.30 | USP32P1 | down | ubiquitin specific peptidase 32 pseudogene 1 |
| ENSG00000154556 | 2.30 | SORBS2 | down | sorbin and SH3 domain containing 2 |
| ENSG00000242770 | 2.29 | CD200R1L-AS1 | down | CD200R1L antisense RNA 1 |
| ENSG00000163083 | 2.29 | INHBB | down | inhibin subunit beta B |
| ENSG00000242375 | 2.22 | - | down | novel transcript |
| ENSG00000160683 | 2.21 | CXCR5 | down | C-X-C motif chemokine receptor 5 |
| ENSG00000186952 | 2.20 | TMEM232 | down | transmembrane protein 232 |
| ENSG00000225434 | 2.15 | LINC01504 | down | long intergenic non-protein coding RNA 1504 |
| ENSG00000213406 | 2.15 | ANXA2P1 | down | annexin A2 pseudogene 1 |
| ENSG00000226937 | 2.15 | CEP164P1 | down | centrosomal protein 164 pseudogene 1 |
| ENSG00000239653 | 2.14 | PSMD6-AS2 | down | PSMD6 antisense RNA 2 |
| ENSG00000213204 | 2.13 | - | down | novel transcript%2C C6orf165-SLC35A1 readthrough |
| ENSG00000260111 | 2.13 | - | down | novel transcript%2C antisense to ST3GAL2 |
| ENSG00000279428 | 2.12 | - | down | TEC |
| ENSG00000286808 | 2.11 | - | down | novel transcript%2C antisense to POLR3E |
| ENSG00000153721 | 2.10 | CNKSR3 | down | CNKSR family member 3 |
| ENSG00000258459 | 2.08 | - | down | novel transcript%2C antisense to CCNB1IP1 |
| ENSG00000253570 | 2.07 | RNF5P1 | down | ring finger protein 5 pseudogene 1 |
| ENSG00000225833 | 2.03 | GS1-594A7.3 | down | uncharacterized LOC104798195 |
| ENSG00000279348 | 2.02 | - | down | TEC |
| ENSG00000225783 | 2.01 | MIAT | down | myocardial infarction associated transcript |
